# Evaluating Changes in Perceived Enjoyment throughout a 12-Week School-Based Exergaming Intervention

**DOI:** 10.3390/children10010144

**Published:** 2023-01-11

**Authors:** Lisa Röglin, Oliver Stoll, Kerstin Ketelhut, Anna Lisa Martin-Niedecken, Sascha Ketelhut

**Affiliations:** 1 Institute of Sport Science, Martin-Luther-University Halle-Wittenberg, 06108 Halle (Saale), Germany; 2 Department of Medical Education and Health, MSB Medical School Berlin, 14197 Berlin, Germany; 3 Institute for Design Research, Zurich University of the Arts, 8005 Zürich, Switzerland; 4 Institute of Sport Science, University of Bern, 3012 Bern, Switzerland

**Keywords:** elementary school, exergaming intervention, long-term psychological effects, moderate-to-vigorous physical activity, physical education

## Abstract

This study assessed whether a high-intensity exergame represents an enjoyable training tool for children in the elementary school setting. Furthermore, it evaluated whether gender, body mass index (BMI), waist-to-height ratio (WHtR), fitness level, weekly physical activity level, and general interest in sports moderate perceived enjoyment during the 12-week intervention. Thirty fifth- and sixth-grade students (10.5 ± 0.7 years; 50% girls) participated in this study. During baseline assessments, anthropometric measurements and a shuttle run test were conducted. Throughout the intervention period, the students participated in 15–20-min exergaming sessions (ES) in the ExerCube twice a week during school hours. Enjoyment was assessed after an ES in weeks 2 and 12. Additionally, enjoyment was evaluated after a physical education (PE) class in week 2. The results reveal no significant changes in enjoyment (*p* = 0.164) over time. The modest changes over time were significantly affected by BMI (*p* = 0.027), WHtR (*p* = 0.007), and weekly activity level (*p* = 0.016). Compared to the PE class, enjoyment was significantly higher during the ES (*p* < 0.001). None of the covariates showed a significant effect. Mean HR during the ES reached 87.1 ± 1.9% of students’ individual maximum HR. In conclusion, the ExerCube provides a promising tool for schools to promote enjoyable moderate-to-vigorous physical activity.

## 1. Introduction

It is widely accepted that a sufficient amount of physical activity (PA) is a component of a healthy lifestyle in children [1]. Several systematic reviews have consistently demonstrated the fundamental benefits of regular PA for body composition, physical fitness, and cardiovascular risk profile in children and adolescents [2,3]. Despite the knowledge of the positive effects of PA, physical inactivity among children and adolescents is a major public health concern [4]. Thus, preventive and sustainable interventions to promote PA among children and adolescents are urgently required. 

In this regard, schools are often considered to play a pivotal role in developing positive PA habits and implementing PA and health programs [5,6] as students spend most of their day at school [7]. Furthermore, school-based PA interventions can easily adapt to local needs and resources, which is crucial for the successful implementation of intervention programs [5]. 

Apart from an appropriate setting, perceived enjoyment during PA is considered a key factor contributing to PA participation and maintenance in childhood [8].

Studies show that enjoyment is the most commonly reported intrinsic motivator for children to engage in PA [9,10]. Research suggests that PA is perceived as more enjoyable when children are encouraged to experiment with a variety of novel activities or with varying familiar exercises [11,12]. In this sense, schools should incorporate PA programs that satisfy the need for novelty and stimulate enjoyment.

An innovative and motivating approach to promote PA and increase enjoyment, especially in children and adolescents reluctant to engage in PA, can be exergames [13,14]. Exergames combine physical exercise with entertaining video gameplay [15] and are therefore considered an important bridge between players’ enjoyment and PA promotion [13,14]. Because of their exercise-promoting features, exergames differ from conventional video games, which are often criticized from a health-science perspective due to their sedentary nature [16,17,18]. Previous studies were able to show that playing exergames increases energy expenditure compared to sedentary behaviors [19,20,21,22,23]. Energy expenditure during exergaming may even be higher than during different forms of PA [24,25].

From a psychological point of view, exergames have been shown to be more enjoyable than traditional exercises, watching television, or playing video games, even in overweight children [26,27]. Research further indicates that exergaming may provoke positive mood states in students [28]. Due to their popularity among youth [29], researchers and pedagogics suggest exergames as a tool to engage students within their own digital culture and promote a healthy and active lifestyle, especially among those who have become less interested in conventional PA approaches [8,30,31,32]. Exergames may provide new and innovative opportunities for the PE curriculum and/or lunchtime, recesses, or after-school-programs [8].

A previous study by Lwin and Malik [33] showed that exergaming incorporated into PE classes combined with health messages has a higher potential to enhance PA-related attitudes and behaviors than regular PE classes, especially in elementary school children. Research further suggests that school-based exergaming interventions can be beneficial in developing children’s musculoskeletal fitness, improving their cardiorespiratory endurance, and enhancing activity time [23,34,35,36].

According to the literature, however, there are substantial differences between exergames currently available on the market [37]. Most exergames only induce light to moderate PA and, thus, do not attain intensity levels required to induce relevant physiological adaptations [38,39,40,41]. Especially, those exergames that are designed for entertainment and/or do not consider general training principles may not attain activity levels high enough to induce health benefits [42,43]. Thus, most exergames may provide joyful, active breaks but should not be recommended to replace PE or school-based PA programs. Furthermore, little is known about the long-term attractiveness of school-based exergaming interventions, especially concerning more physically demanding exergames.

It is questionable whether exergames are able to provide enjoyable exercise approaches in the school setting over time or whether enjoyment fades as soon as the exergaming intervention becomes a routine.

The present study assessed a mixed-reality exercise setting, the ExerCube [44], which provides both an attractive and effective exergame experience for different target groups [45,46]. 

According to earlier studies in adults, the ExerCube provides a high-intensity exercise stimulus [47] yielding higher scores for flow, motivation, and enjoyment compared to other exercise protocols regardless of gender and individual performance level [45]. Based on these results, the present study was conducted to assess whether this exergame also provides a physiologically relevant but enjoyable exercise experience for children during a school-based intervention.

This study aimed to determine whether perceived enjoyment while exercising regularly in the ExerCube changes over time and whether gender, body mass index (BMI), waist-to-height ratio (WHtR), and maximal oxygen consumption (VO_2_max) as well as weekly activity level and general interest in sports moderate the possible changes. To provide a better understanding of the assessed ExerCube enjoyment scores, the study further compared the students’ perceived enjoyment during an exercise session in the ExerCube with that during a regular PE class.

## 2. Materials and Methods

### 2.1. Research Setting and Participants

Thirty fifth- and sixth-grade students (10.5 ± 0.7 years; 15 girls) from an inner-city elementary school in Berlin (Germany) participated in this study. According to an a priori power analysis (G*power, version 3.1.; Heinrich Heine University, Düsseldorf, Germany), a sample size of 15 participants would provide sufficient power (0.8) to detect differences. A large effect size of 0.4 (Cohen’s f) and an alpha level of 0.05 were assumed.

The school’s principal, teachers, and parents were provided with detailed study information. Written parental consent was obtained before participation in the study. Only students who provided a written informed parental consent form were eligible to participate. Students with health conditions that did not allow unrestricted PA engagement were excluded.

During the intervention period, the students participated in the regular PE classes twice a week (in total, 135 min), taught by a certified full-time PE teacher and held in the school’s gym. The exergaming sessions (ES) took place in the ExerCube, which was set up by Sphery Ltd. (Au, Switzerland). in a room located close to the students’ classrooms (multi-purpose rooms).

The study was conducted in accordance with the Helsinki Declaration and approved by the Research Ethics Board of the Medical Center Berlin (2020-09-RK1).

### 2.2. Procedure

The study design consisted of a baseline examination followed by an intervention period lasting three months. During the baseline examination, anthropometric measurements, including body mass, height, and waist circumference, were assessed to calculate the BMI (=body mass (kg)/height (m)^2^) [48] and WHtR (=waist circumference (cm)/height (cm)) [49]. Habitual PA and sports interest were assessed using selected items of a validated questionnaire (Motorik-Modul-Physical-Activity-Questionnaire for children and adolescents (MoMo-PAQ) [50]. Furthermore, the students completed a 20 m shuttle run test to estimate VO_2_max [51]. Trained study staff conducted all measurements under the same conditions on the school’s premises.

Throughout the three-month intervention period, the students completed two ES per week, with each session lasting about 15–20 min. The ES were held during school time but outside PE classes to guarantee that the intervention would not be conducted at the expense of the students’ available exercise time. Together with the respective class teachers, timetables for each student were developed, supporting regular and consistent participation and a smooth process. However, students still had the option to withdraw from the intervention. Trained study staff supervised the students during the ES. Throughout the intervention period, perceived enjoyment during the ES was assessed twice (week two and week 12) using the “Physical Activity Enjoyment Scale” (PACES). Additionally, the same group of students were asked to complete the PACES at the end of a randomly selected PE class in the second week. The PE class consisted of various free-choice sports and movement games (no exergames as they are generally not part of the school’s PE curriculum), which are part of the typical content of the curriculum. There were no graded physical performance tests in this lesson. The order in which the students completed the questionnaires (after the ES and the PE class) was randomized and counterbalanced.

After the intervention period, we asked the teachers and study staff for feedback using short, structured interviews.

### 2.3. The Exergame Setting

**Figure 1:**
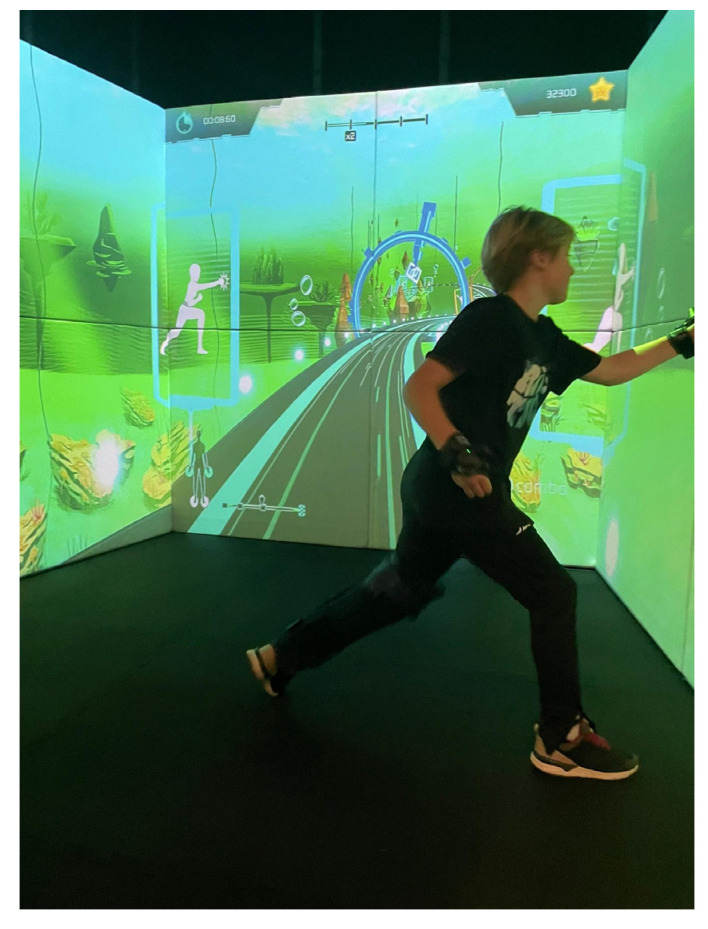
Student playing “Sphery Racer” in the ExerCube by Sphery Ltd. (Au, Switzerland) © Anna Lisa Martin-Niedecken.

The ExerCube by Sphery Ltd. (Au, Switzerland) (Figure 1) is an exergame setting shaped as an open cube-like trapeze measuring 9 m^2^. The three walls of the ExerCube project the virtual game scenario and provide an interface for generating in-game actions [44]. In contrast to many other exergames, which are played in front of a screen using a controller, the ExerCube allows the players to fully immerse themselves in and interact with the virtual game scenario through whole-body movements. 

In the present study, the students played the single-player game experience “Sphery Racer,” consisting of a science-fiction inspired virtual racing track. The player’s task is to navigate an avatar on a hoverboard through five game levels. The avatar is controlled by different movement tasks such as jumps, punches, or squats. To score points, the movement tasks must be performed as precisely as possible within a restricted time. Thus, the game challenges the player not only coordinatively but also conditionally and cognitively [11]. Throughout the game, the game’s challenge is continuously tailored to the physical and mental performance of the player.

The player’s movements are tracked and incorporated into the game via the HTC Vive tracking system, consisting of two wrist-worn and two ankle-worn trackers. Calibration at the beginning of each exergaming session ensures that the game is optimally adjusted to the player’s body size. HR is continuously recorded during the game utilizing an HR monitor (Polar Electro Oy, Kempele, Finland).

More details about the ExerCube are provided by Martin-Niedecken et al. [44,52].

### 2.4. Measures

#### 2.4.1. Anthropometry

Body mass was determined to the nearest 0.1 kg while the students wore light sports clothes. Height was obtained without shoes to the nearest 0.5 cm. Waist circumference was assessed to the nearest 0.5 cm at the umbilical line while the students were standing [53]. All measurements were conducted using standardized measuring equipment. BMI and WHtR were calculated for each student.

#### 2.4.2. Enjoyment

A modified version of the PACES [54] consisting of 16 bipolar statements was used to measure perceived enjoyment during the ES and PE class. On a five-point bipolar scale, the students rated how they felt about the exercise they had just performed. The total score of the questionnaire ranged between 16 and 80, with higher scores indicating higher enjoyment. A mean score was calculated for each session. Before answering the questionnaire, the students received standardized instructions and were asked to complete it as truthfully as possible. The PACES has been widely used in PA and exergaming environments and is appropriate for children [55]. Furthermore, it has been validated as a reliable and valid measuring instrument, with good internal consistency between 0.92 and 0.93 [54].

#### 2.4.3. Self-Reported Habitual Physical Activity and Interest in Sports

Two items of the German version of the MoMo-PAQ [50] for students were selected to assess self-reported weekly PA and students’ general interest in sports. The students were asked to rate “how interested are you in sports?” using a five-point bipolar rating scale from “not interested” to “very interested” (MoMo-PAQ, section: general PA, item five). Additionally, the students were asked to state the number of days (0–7) they were physically active for more than 60 min in a typical week (MoMo-PAQ, section: general PA, item four).

#### 2.4.4. Teacher and Study Staff Feedback

After the intervention period, short, structured interviews were conducted with the study staff and the respective class teachers. The interviews consisted of three questions assessing their perception of students’ interest, engagement, and motivation during the ES. The interviews lasted 3–5 min.

#### 2.4.5. Aerobic Fitness

The level of aerobic fitness was assessed using the multistage 20 m shuttle run test. The shuttle run test is a reliable and standard method to predict VO_2_max both in children and adults [51]. The students were required to run back and forth between two lines, set 20 m apart while keeping a predetermined pace. The pace was set with audio signals emitted at specific frequencies.

The first stage started at 8.5 km/h and was increased each minute/stage by 0.5 km/h. The students were instructed to keep running as long as possible, completing as many shuttles and stages as possible. The test was over when the student was not able to reach the appropriate lines in the allotted time two consecutive times or when they gave up due to fatigue. 

In order to predict aerobic fitness, the number of fully completed shuttles and stages, as well as the maximal shuttle running speed, were recorded for each student. Shuttle running speed and age were used to predict VO_2_max according to the model developed by Leger et al. [51]:

VO_2_max = 31.025 + 3.238 × maximal shuttle run speed − 3.248 × age + 0.1536  × maximal shuttle run speed × age (1)

#### 2.4.6. Heart Rate

HR was continuously recorded during the ES using a HR monitor (Polar Electro Oy, Kempele, Finland). Mean HR (HRmean) was calculated for each student by summing up HRmean of all ES. To determine the percentage of HRmax achieved during the ES, individual HRmax was estimated using the formula (HRmax = 208 − 0.7 × age) of Tanaka et al. [56].

### 2.5. Data Analysis

IBM SPSS Statistics for Windows, Version 27.0 (IBM Corp. Released 2020, Armonk, NY, USA) was used to analyze the collected data. The significance level adopted was *p* < 0.05. All results are presented as means ± standard deviation. 

A regression analysis was conducted to analyze the relationship between the dependent variable “perceived enjoyment” and the independent variables gender, BMI, WHtR, weekly activity level, general interest in sports, and VO_2_max during an ES at the beginning of the intervention. 

Repeated measures ANOVAs were performed to examine differences in perceived enjoyment over time and differences in perceived enjoyment between the ES and the PE class. Additionally, repeated measures ANCOVAs using gender, BMI, WHtR, weekly activity level, general interest in sports, and VO_2_max as covariates were conducted. 

Because the sample of this study was rather small, normal distribution was assessed in advance using the Kolmogorov−Smirnov test. The findings suggest that normal distribution was violated for a subset of variables/factor combinations. However, as several studies demonstrate the robustness of ANOVA against violations of the normal distribution assumption [57,58], we felt it safe to run the reported analyses to examine possible interactions. Nevertheless, to further corroborate the robustness of the effects, we additionally ran the analyses with a non-parametric Wilcoxon signed-rank test. These analyses reconfirmed the results.

To determine effect size, Cohen’s f (f) was calculated (f > 0.1: small effect; >0.25: medium effect; >0.4: large effect) [59].

## 3. Results

All students completed the initial examination. The session attendance rate for the ES was 97%. Three students were unable to take part in the survey in the second week of the intervention and were therefore excluded from the analysis. No adverse events occurred throughout the intervention period. The students’ descriptive and anthropometric measures are shown in Table 1.

According to the age- and sex-specific BMI percentiles from Coners et al. [48], nine students (three girls) could be defined as obese (BMI percentiles ≥ 95), and two students (two girls) could be defined as overweight (BMI percentiles > 85). For the WHtR, eight students (two girls) reported values within the overweight range (WHtR cutoff of 0.5 [49]).

The regression analysis revealed no significant relationship between perceived enjoyment and gender (*p* = 0.763), BMI (*p* = 0.113), WHtR (*p* = 0.231), weekly activity level (*p* = 0.967), general interest in sports (*p* = 0.188), and VO_2_max (*p* = 0.885) during an ES (Table 2).

**Figure 2:**
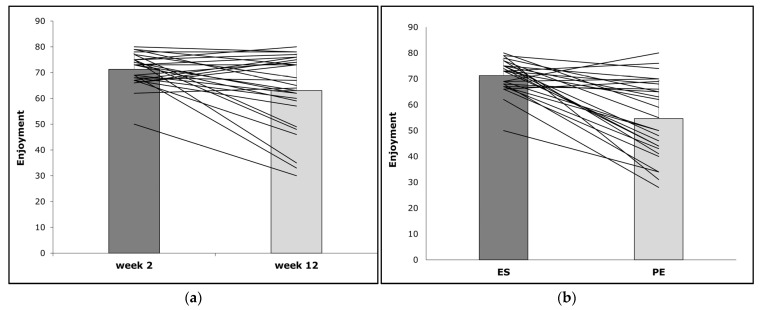
(**a**) Perceived enjoyment during an exergaming session in weeks two and 12; (**b**) Perceived enjoyment during an exergaming session and a PE class in week two. In both figures, the bars show the mean Physical Activity Enjoyment Scale (PACES)-scores. The lines represent individual changes.

The differences in perceived enjoyment over time and between an ES and a PE class are shown in Figure 2a,b. The mean PACES score during the ES in week two was significantly higher than during the PE class in the same week (71.3 ± 6.3 versus 54.6 ± 14.7; *p* < 0.001; f = 0.705). None of the analyzed covariates (gender (*p* = 0.94, f = 0.000), BMI (*p* = 0.152, f = 0.100), WHtR (*p* = 0.461, f = 0.027), weekly activity level (*p* = 0.957, f = 0.000), general interest in sports (*p* = 0.684, f = 0.008), and VO_2_max (*p* = 0.389, f = 0.037) showed a significant effect on the differences. Furthermore, the analysis revealed no significant differences in perceived enjoyment (*p* = 0.164) after two and 12 weeks of intervention. The mean PACES score changed from 71.3 ± 6.3 in week two to 62.4 ± 14.2 in week 12. A small effect size (f = 0.073) could be identified for the differences over time. According to the ANCOVA, the differences in perceived enjoyment between weeks two and 12 were significantly affected by BMI (*p* = 0.027, f = 0.227), WHtR (*p* = 0.007, f = 0.326) and weekly activity level (*p* = 0.016, f = 0.265). Gender (*p* = 0.986, f = 0.000), general interest in sports (*p* = 0.825, f = 0.002), and VO_2_max (*p* = 0.109, f = 0.124) showed no significant effect on the differences in perceived enjoyment over time.

During the ES, the students reached an average HRmean of 174.9 ± 3.9 bpm corresponding to 87.1 ± 1.9% of their individual HRmax.

In the feedback interviews, the teachers and study staff consistently reported high levels of interest, motivation, and engagement of most students throughout the ES. Three of the four teachers mentioned that even students who were usually unmotivated to participate in PE were highly interested in the ES. 

## 4. Discussion

The purpose of this study was to assess the perceived enjoyment throughout a 12-week school-based exergaming intervention in elementary school children. Additionally, the study determined whether gender, BMI, WHtR, weekly activity level, general interest in sports, and VO_2_max influenced perceived enjoyment over time. Furthermore, perceived enjoyment between an ES and a normal PE class was compared while considering the aforementioned covariates. Based on the present results, the ES represent an enjoyable and vigorous exercise both for boys and girls irrespective of their physical fitness level. Thus, this exergame may present an innovative and engaging tool to promote PA throughout the school day. 

Notably, the mean ExerCube enjoyment score did not significantly decrease throughout the 12-week intervention period, although the exercise intensity was relatively high, reaching 87.1 ± 1.9% of HRmax. This corresponds to vigorous intensity PA according to the guidelines for exercise testing and prescription of the American College of Sports Medicine [60]. Interestingly, when we looked at the individual results (Figure 2a), we were able to detect both increases and decreases in perceived enjoyment over time. Even when considering the students’ characteristics (e.g., gender, BMI, WHtR), no clear pattern can be identified. Thus, we cannot make any assumptions about possible reasons for these differences. Despite this, the ExerCube presents a tool to implement joyful, high-intensity PA and keeps most students engaged over a long period of time. The findings are relevant as a decrease in PA can be observed especially at a prepubertal age and in early puberty, which is attributed to a lack of perceived PA enjoyment [61].

The results are in accordance with previous research by Sun [62], who reported high levels of perceived enjoyment during a four-week school-based exergaming intervention in elementary school children using eight different exergames (e.g., Nintendo Wii, Dance Dance Revolution, or XrBoards). However, the exercise did not reach a moderate to vigorous intensity in this study. Fu et al. [49] could even show a significant increase in students’ perceived enjoyment during a 12-week classroom-based exergaming intervention. Unfortunately, the authors did not report the exercise intensities of the applied exergames. Lau et al. [63] could also observe an increase in enjoyment throughout a 12-week after-school-hour exergaming intervention in 8–11-year-old children compared to a control group. However, the differences did not achieve statistical significance. Additionally, this study did not examine the intensity of the 60 min exergaming intervention.

Because studies suggest that enjoyment is associated with higher PA engagement [64], enjoyable exergames could possibly influence future PA behavior. Furthermore, enjoyment has been identified as an underlying factor in children and adolescents for maintaining their engagement in both PA and PE [65,66,67]. However, there is still a necessity to further address the influence of regular school-based exergaming on PA behavior and general PA enjoyment.

The sustained enjoyment level throughout the exergaming intervention can be explained by the innovative setup and game design. The ExerCube provides an immersive experience, allowing the players to interact with the audio-visual gaming scenario through whole-body movements. According to Warburton et al. [68], such an immersive experience diverts the players’ attention from the physiological cues and increases enjoyment. Therefore, exergaming may not be primarily experienced as exercise, but as a form of entertainment [69]. The interactive character of exergames may further distract from negative thoughts about PA [70,71]. Therefore, exergames could help to motivate particularly those students who are unwilling to engage in more conventional PA approaches. 

A further explanation for the sustained experience of enjoyment throughout the exergaming intervention could be related to its digital environment and state-of-the-art technology. Because young people devote considerable amounts of their leisure time to screen-based activities [72], the integration of exergames into the children’s school routine may present an enjoyable and culture-appropriate contrast to the primarily analog curriculum. Working with, rather than against, preferred digital leisure routines at school can be a promising approach to promote PA in children. This is supported by the finding that the ES was significantly more enjoyable than the PE class with none of the covariates (gender, BMI, WHtR, weekly activity level, general interest in sports and VO_2_max) showing a strong effect. Only two students rated the PE class as more enjoyable than the ES (Figure 2b). 

The findings are in accordance with a previous study by Vernadakis et al. [26], who reported no significant differences between normal-weight and overweight children in their enjoyment of exergames, traditional physical activities, and sedentary video games. In this study, the exergame was the most enjoyable activity, in both normal weight and overweight children.

The appeal of the exergaming intervention in the present study is further supported by the fact that there were no dropouts. 

High adherence levels were also shown by Sheehan et al. [73] during a six-week school-based exergaming program. In this qualitative study, the teachers reported that students’ enthusiasm for the intervention was high, resulting in sustained engagement. Furthermore, the teachers reported that the students felt proud to have an exergaming opportunity in their school. 

According to feedback interviews, the teachers of the present study also reported high levels of interest during the exergaming intervention, even among students, who were usually unmotivated to participate in PE. Low dropout rates were also observed by Finco et al. [74], who further discovered an increasing interest of students in learning more about different sports and a healthy lifestyle during a school-based exergaming intervention.

Interestingly, Madsen and colleagues [75] found children to have higher dropout rates when exergaming at home compared to exergaming interventions at school. This underlines that an appropriate setting and intervention design are of great relevance for successfully implementing PA interventions in children. Simply owning an exergame is not enough to promote PA and provide a public health benefit in children [76].

Another explanation for the sustained enjoyment is the ability of the ExerCube to adjust the game’s challenge according to the player’s physical and cognitive performance. This guarantees an optimal balance between the game-related challenge and their skills, as well as between the movement tasks and the player’s fitness. Consequently, the challenge is neither too easy nor too difficult, allowing them to experience success. This is of relevance as previous research has shown that perceived physical competence is associated with intrinsic motivation [77] and enjoyment in PA [72] and PE [78,79]. An adaptive game design, gamification, and gratification can help to evoke perceived physical competence. 

According to Csikszentmihalyi’s flow theory, this perceived balance between challenge and skills is a precondition for the experience of flow, which is described as the feeling of being completely focused on a particular activity [80]. Referring to “GameFlow” research in the context of video gaming [81] and exergaming [82], the flow experience is regarded as an important determinant of enjoyment in gaming [81,83,84]. The ExerCube already yielded significantly higher flow scores in adults than traditional training approaches, as shown in recent studies by Röglin et al. [45] and Martin-Niedecken and Schättin [85].

In the present study, the regression analysis revealed no relationship between perceived enjoyment and gender, BMI, WHtR, weekly activity level, general interest in sports, and VO_2_max during the ES. Therefore, it can be expected that the ExerCube adapts to the students’ preconditions and represents an attractive exercise for different target groups. Regarding gender, the findings contrast a study by Sun [62], who reported that boys rated the exergaming experience as more enjoyable than girls. This is in accordance with research in video gaming, suggesting that video games are less attractive to girls compared to boys [86,87]. The fact that gender had no effect on perceived enjoyment during the ES is an important finding as girls are less physically active than boys [88,89]. Furthermore, girls are more reluctant to perform PA at higher intensities [90], and generally score lower on enjoyment in PE class [78]. The ExerCube is an exercise tool that seems to reach boys and girls alike and thus can help tackle gender disparities. Furthermore, neither general interest in sports nor VO_2_max moderated the minor differences in perceived enjoyment over time. The findings underline the appeal of the exergame experience for students regardless of their gender, individual fitness level, and passion for sports. In contrast, BMI, WHtR, and weekly activity level significantly affected the modest differences in perceived enjoyment over time. Thus, further research should address how exergames can be tailored and constantly developed to guarantee persistent engagement in different target groups. Similarly to normal video games, exergames could also benefit from including regular updates or expansions to the game to continuously provide new opportunities for exploration.

Furthermore, previous research shows that cooperative and competitive exergaming conditions result in greater enjoyment and higher motivation compared to single-player conditions [91]. Thus, further research should address if the cooperative and competitive game modes may be an approach to sustain perceived enjoyment even longer.

However, an interesting feature of the single-player mode is the fact that it adheres to social distancing and hygiene measures. This could be particularly applicable in times of pandemics (e.g., COVID-19). Exercise interventions in the ExerCube can be maintained even if it is not allowed to perform group exercises or PE classes. This is of relevance, as research revealed a substantial decrease in PA during the COVID-19 pandemic, which may negatively impact children’s and adolescents’ physical and mental health [92,93].

### Limitations

The following limitations must be discussed when interpreting the results of the study. First, the students had no experience with the ExerCube before this study. All students, however, participated regularly in the PE classes. Thus, the ES could have benefited from a novelty effect. Recent research discovered that inexperienced players achieve higher levels of enjoyment than experienced players [94]. However, at least during the 12-week intervention period, perceived enjoyment did not decrease significantly.

Second, only one random PE class was chosen as a comparison condition. It is to be expected that perceived enjoyment may have been affected by the lessons’ content and the respective exercise intensity, and thus could vary between sessions. However, the students’ exercise intensity during PE class was not assessed. Furthermore, differences in the duration of the two exercise conditions could have affected perceived enjoyment.

Third, individual HRmax of the students was calculated using the formula of Tanaka et al. [56]. This calculation only presents a rough estimation of the actual HRmax and is not specific for the study’s sample.

Another limitation to discuss is the duration of the intervention period. Due to holidays and other school-related conditions, the intervention took place throughout a 12-week period. Therefore, it is not clear if the results hold true for longer intervention periods. Furthermore, it has not been investigated whether the ES affected general PA enjoyment and future PA behavior.

Lastly, the target group of this study consisted of fifth- and sixth-grade students. It cannot be assumed that the results also apply to other grades. Furthermore, the sample size of this study was rather small, and the study design does not include a control group.

## 5. Conclusions

The ExerCube provides a promising tool for elementary schools to promote enjoyable PA among boys and girls with different preconditions during the school day. Therefore, this exergame can be applied as an appropriate alternative or supplement to traditional exercise approaches to promote active routines at school and, thus, healthy lifestyle behaviors among children. Future studies are required to assess the effects of different exergaming intervention designs on perceived enjoyment among different target groups. Furthermore, it should be evaluated whether and to what extent regular school-based exergaming sessions affect PA behavior and PA enjoyment in general.

## Figures and Tables

**Table 1:** Subject’s characteristics.

Item	Total (*n* = 27)	Female (*n* = 14)	Male (*n* = 13)
Height (cm)	148.4 ± 9.2	150.1 ± 10.9	146.5 ± 6.4
Body mass (kg)	46.2 ± 12.8	46.0 ± 14.5	46.5 ± 10.6
Waist circumference (cm)	67.6 ± 10.1	64.8 ± 8.5	70.8 ± 10.8
BMI (kg·m^−2^)	20.7 ± 4.2	20.0 ± 4.3	21.5 ± 3.9
WHtR	0.45 ± 0.06	0.43 ± 0.04	0.48 ± 0.06
VO_2_max (mL/kg/min)	44.70 ± 3.5	44.70 ± 3.5	44.74 ± 3.4
Physical activity level (days/week with over 60 min)	3.6 ± 2.2	3.1 ± 2.1	4.2 ± 2.2
General interest in sports (1 = no interest; 5 = very interested)	4.1 ± 0.9	4.0 ± 1.1	4.3 ± 0.8

Data are mean ± SD values. Abbreviations: BMI = Body mass index; VO_2_max = Maximal oxygen consumption; WHtR = Waist-to-height ratio.

**Table 2:** Effects of gender, BMI, WHtR, weekly activity level, general interest in sports, and VO_2_max on perceived enjoyment during the exergaming session.

Variable	Unstandardized Coefficient	Standardized Coefficient	Std. Error
Constant	61.445		
Gender	0.881	0.070	2.876
BMI (kg·m^−2^)	−0.066	−0.164	0.119
WHtR	54.133	0.534	43.837
Physical activity level (days/week with over 60 min)	−0.024	−0.008	0.573
General interest in sports (1 = no interest; 5 = very interested)	2.026	0.313	1.487
VO2max (mL/kg/min)	−0.077	−0.040	0.527
R^2^	0.315		
Adjusted R^2^	0.110		
F (df = 6;20)	1.535		

Abbreviations: BMI = Body mass index; VO_2_max = Maximal oxygen consumption; WHtR = Waist-to-height ratio.

## Data Availability

The data that support the findings of this study are available on request from the authors.
